# Microwave-Synthesized Platinum-Embedded Mesoporous Silica Nanoparticles as Dual-Modality Contrast Agents: Computed Tomography and Optical Imaging

**DOI:** 10.3390/ijms20071560

**Published:** 2019-03-28

**Authors:** Chia-Hui Chu, Shih-Hsun Cheng, Nai-Tzu Chen, Wei-Neng Liao, Leu-Wei Lo

**Affiliations:** 1 Institute of Biomedical Engineering and Nanomedicine, National Health Research Institutes, Zhunan, Miaoli County 35053, Taiwan; 981012@nhri.org.tw (C.-H.C.); smallgi2002@gmail.com (S.-H.C.); 970730@nhri.org.tw (W.-N.L.); 2 Institute of New Drug Development, China Medical University, Taichung 40402, Taiwan; ohnonancy@gmail.com

**Keywords:** mesoporous silica nanoparticles (MSNs), dual-modality contrast agents, naked platinum nanoparticles (nPtNPs), microwave synthesis, computed tomography (CT)

## Abstract

Nanoparticle-based imaging contrast agents have drawn tremendous attention especially in multi-modality imaging. In this study, we developed mesoporous silica nanoparticles (MSNs) for use as dual-modality contrast agents for computed tomography (CT) and near-infrared (NIR) optical imaging (OI). A microwave synthesis for preparing naked platinum nanoparticles (nPtNPs) on MSNs (MSNs-Pt) was developed and characterized with physicochemical analysis and imaging systems. The high density of nPtNPs on the surface of the MSNs could greatly enhance the CT contrast. Inductively coupled plasma mass spectrometry (ICP-MS) revealed the MSNs-Pt compositions to be ~14% Pt by weight and TEM revealed an average particle diameter of ~50 nm and covered with ~3 nm diameter nPtNPs. To enhance the OI contrast, the NIR fluorescent dye Dy800 was conjugated to the MSNs-Pt nanochannels. The fluorescence spectra of MSNs-Pt-Dy800 were very similar to unconjugated Dy800. The CT imaging demonstrated that even modest degrees of Pt labeling could result in substantial X-ray attenuation. In vivo imaging of breast tumor-bearing mice treated with PEGylated MSNs-Pt-Dy800 (PEG-MSNs-Pt-Dy800) showed significantly improved contrasts in both fluorescence and CT imaging and the signal intensity within the tumor retained for 24 h post-injection.

## 1. Introduction

Nanoparticles (NPs)-based imaging contrast agents have emerged as a vigorous research area with agents such as iron oxide for magnetic resonance imaging (MRI) [1,2,3], quantum dots for optical imaging [4,5], and gold NPs for X-ray computed tomography (CT) [6,7,8] being developed. Although NPs can be modified with specific targeting molecules to enhance their delivery to tumors and, therefore, increase tumor contrast, a high percentage of administered NPs will nonetheless accumulate in tissues/organs other than the targeted tumor. Substantial amounts of NPs residing in tissues/organs that do not undergo further biodegradation or excretion could cause unwanted cytotoxicity and other side effects [9,10,11,12,13,14,15]. Thus, defining the characteristics of synthetic NPs that govern their biodegradation and/or excretion in vivo is a prerequisite for the successful application of such NPs as imaging contrast agents in clinical applications. In this study, we aimed to develop dual-modality NP-based contrast agents for tumor imaging. Orthotopic tumor models [16,17], such as hepatomas, have been established for the validation of imaging contrast in the respective imaging modality. A single NP that possesses dual imaging modalities should provide a comprehensive diagnostic nanoplatform for cancer prognosis [18,19,20,21,22,23,24]. 

Mesoporous silica (MPS) materials have generated considerable interest since they were first synthesized by Beck et al. in 1992 [25]. MPS has many advantages, such as a large surface area (approximately 1000 m^2^ g^−1^), large pore volume (near 1.0 cm^3^ g^−1^), a highly ordered pore structure, and adjustable pore size (2.0–30 nm). These properties have led to widespread and interesting applications in the chemical catalysis and biomedical fields [26,27,28,29,30,31,32,33,34]. Recently, the particle form of nano-sized MPS materials (MSNs) has been vigorously studied as vehicles for the targeted delivery and controlled release of drugs.

Platinum, which has high X-ray absorption (X-ray absorption coefficient of Pt: 6.95 cm^2^ g^−1^ at 50 keV), can serve as a potential CT contrast agent [35]. We developed a one-pot synthesis for preparing small-sized naked platinum nanoparticles (nPtNPs) on amine-modified mesoporous silica NPs via a microwave irradiation reduction process. Aminosilane molecules exhibit high basicity and, thus, can easily undergo protonation to carry a great quantity of positive charges. These positive charges can produce strong electrostatic attractions with PtCl^6−^ ions. After microwave irradiation, the use of primary amine ligands as a stabilizer had a protective effect in the growth of water-dispersible nPtNPs with a small size on the surface of MSNs. We obtained a high density (14%) of nPtNPs on the surfaces of the MSNs (MSNs-Pt), as indicated by ICP-MS (2.8 mg Pt per 20 mg MSNs-Pt). This high density of nPtNPs can greatly enhance CT contrast and minimize the necessary injection dosage. On the other hand, it is well known that nPtNPs exhibit nanozyme-like activity, including peroxidase, catalase, superoxide dismutase, and oxidase properties. nPtNPs have received more attention in the field of catalysis and medicine due to their excellent catalytic activity. It was demonstrated that nPtNPs possess intrinsic catalytic and biological activities, which strongly depend on the environmental pH [36]. Following these results, nPtNPs-MSN nanocomposite was subsequently synthesized, which exhibits high catalytic activity confirmed by the oxidation of 3,3′,5,5′-tetramethylbenzidine [37]. However, the low stability of nPtNPs and tendency for aggregation have been substantial problems. Surface modification with biomolecules and synthetic ligands has been used to stabilize nPtNPs; for instance, chitosan to stabilize nPtNPs against aggregation while enhancing their oxidase-like activity [38]. One group has proposed a simple label-free DNA detection by amplifying the catalytic signal of nPtNPs inside MSNs as a smart reporter [39]. A facile and simple method for the preparation of nPtNP-decorated two-dimensional metal-organic framework (MOF) nanocomposites was developed by Chen’s research group. The obtained nPtNPs/Cu-TCPP(Fe) hybrid nanosheets exhibited increased peroxidase-like catalytic activity due to the synergistic effects of the unique structure [40].

To meet the requirements of intravenous injection by evading the reticuloendothelial system (RES), sub-100 nm NPs should be designed and fabricated. Recently, Hyeon et al. reported the fabrication of uniform mesoporous dye-doped silica NPs immobilized with multiple magnetite nanocrystals on the surface (designated as Fe_3_O_4_-MSNs), as well as their application in simultaneous MRI, fluorescence imaging, and as a drug delivery vehicle [21]. Herein, we report dual-modality MSNs created by exploiting different topological domains containing a large number of nPtNPs on the outermost surface for X-ray CT contrast and a NIR contrast agent (DyLight 800) loaded in the nanochannels for optical imaging (OI). To the best of our knowledge, we are the first group to employ the microwave method to reduce naked nPtNPs on an MSN template and to exploit this integration to enhance CT signals. Additionally, the MSNs retained the ability to load anticancer drugs for delivery. In this paper, we demonstrate in vivo optical and CT imaging using mice bearing MDA-MB-231 breast cancer model.

## 2. Results

### 2.1. Synthesis of Dual-Modality MSNs Containing nPtNPs on the Outermost Surface for CT Contrast and NIR Probe (Dy800) in the Nanochannels for OI

**Figure 1:**
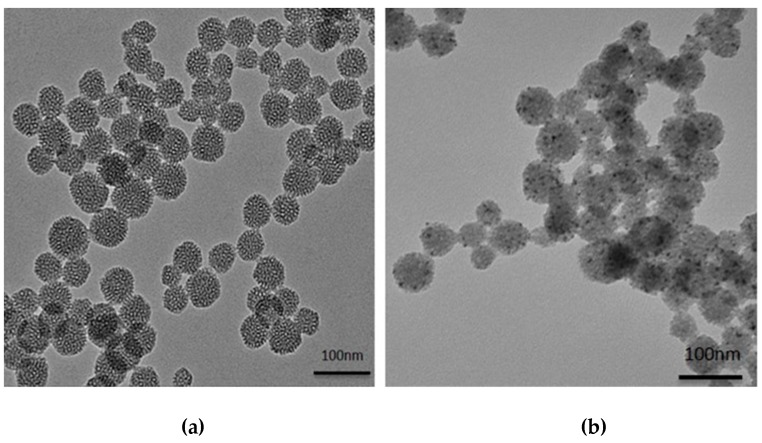
TEM images of (**a**) large-pore mesoporous silica nanoparticles (MSNs) with a diameter of 50 nm and (**b**) the microwave-synthesized MSN-Pt nanoparticles.

Large-pore MSNs were synthesized; a TEM image of these MSNs is shown in Figure 1a. Using *n*-octane as a swelling agent to expand the inner micelle space, we observed that the as-synthesized MSNs possessed both large pore diameters (~5.0 nm) and well-ordered pore structures. The large-pore MSNs had sphere-like shapes and small sizes with average particle diameters of approximately 50 nm. We modified the primary amine group in the channels and on the surface of MSNs. Before the pores were extracted, the adsorption of Pt^4+^ ions to the surfaces of the aminosilane-modified silica nanoparticles was achieved using electrostatic attractions between the positively charged surface of the amine-modified silica and the negative charge of the PtCl^6−^ ions. Using microwave irradiation, the PtCl^6−^ ions were fully converted to Pt^0^ NPs on the MSN surface. After microwave reduction, TEM images of the nPtNP-embedded MSNs were collected, as shown in Figure 1b. The size of the nPtNPs was approximately 3 nm, and the weight percentage of Pt on the outer surface of MSNs was calculated to be 14 % using ICP-MS. The naked nPtNPs on the MSNs were easily modified by thiol groups. Thiol-terminated polyethylene glycol (PEG) could be conjugated to the nPtNPs to enhance their dispersibility in bio-media and to prevent protein adsorption onto the MSNs-Pt NPs. Moreover, many target peptides that have thiol side chains, such as cysteine, can also be attached to the MSNs-Pt. The NIR fluorescence dye used for the OI modality, Dy800, was encapsulated into the nanochannels of the MSNs-Pt. The process involved using the Dy800-NHS ester to react with aminosilane, followed by mixing with the pore-extracted MSNs-Pt to yield MSNs-Pt-Dy800.

**Figure 2:**
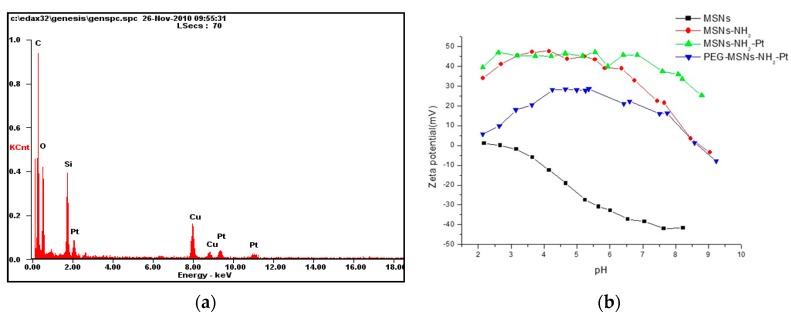
Nanoparticles (NPs) characterization. (**a**) The energy-dispersive X-ray (EDX) spectrum of the MSNs-Pt. (**b**) The zeta potential measurements of MSNs-NH_2_, MSNs-NH_2_-Pt, and PEG-MSNs-Pt NPs.

Figure 2a presents the energy-dispersive X-ray spectrum (EDX) of the MSNs-Pt. We observed three peaks at 2, 9.2, and 11 KeV that represent the composition of Pt and the peak from 1.9 KeV represents the Si in the spectrum. Pt elements were clearly observed in the spectrum, providing evidence for the incorporation of the nPtNPs onto the MSNs. Figure 2b presents the zeta potentials of the amine-modified MSNs (MSNs-NH_2_), MSNs-NH_2_-Pt, and PEG-modified MSNs-Pt (PEG-MSNs-Pt) at pH values ranging from 2 to 10. The zeta potential of the MSN-NH_2_-Pt was lower than that of the MSNs-NH_2_ in the pH range from 7~10 because of the positively charged functional groups conjugated to the surface and because the Pt:aminopropyl groups on the surfaces of the MSNs were bonded with the Pt NPs. After PEG modification, the MSNs-Pt-PEG possessed a neutral surface charge.

**Figure 3:**
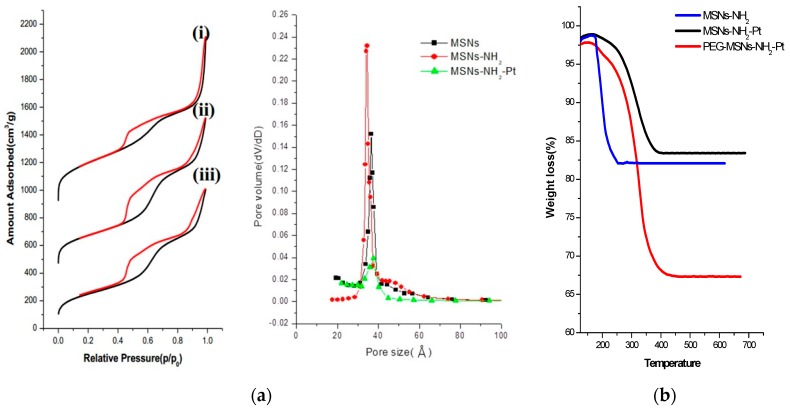
(**a**) The nitrogen adsorption–desorption isotherms and pore size distributions of (i) MSNs, (ii) MSNs-NH_2_ and (iii) MSNs-NH_2_-Pt. (**b**) The thermogravimetric analysis (TGA) measurement of MSNs-NH_2_, MSNs-NH_2_-Pt, and PEG-MSNs-Pt. The PEG-MSNs-Pt exhibited a 24.5% greater weight loss than did the MSNs-Pt.

The surface areas and pore size distributions of the MSNs were characterized using the Brunauer–Emmett–Teller (BET) and Barrett–Joyner–Halenda (BJH) methods. The nitrogen adsorption–desorption plot of the MSNs (Figure 3a-i) exhibited a typical type IV isotherm and a pore size distribution centered at 3.6 nm. The surface area and pore volume were calculated to be 1073.63 m^2^ g^−1^ and 0.88 cm^3^ g^−1^, respectively. The surface areas of the MSNs-NH_2_ and MSNs-Pt decreased to 844.15 m^2^ g^−1^ and 649.77 m^2^ g^−1^, respectively (as shown in Figure 3a-ii,a-iii). The majority of the Pt NPs were reduced on the surface of the MSNs to leave sufficient surface area for drug delivery, catalysis, and other applications. Thermogravimetric analysis (TGA) was employed to identify the surface modification. Figure 3b indicates that PEG accounted for 24.5% of the total mass of the PEG-MSNs-Pt NPs. This high percentage of PEG coating the MSNs can provide a longer circulation time for in vivo optical/CT imaging.

### 2.2. In Vitro Optical and CT Images of Dual-Modality MSNs

**Figure 4:**
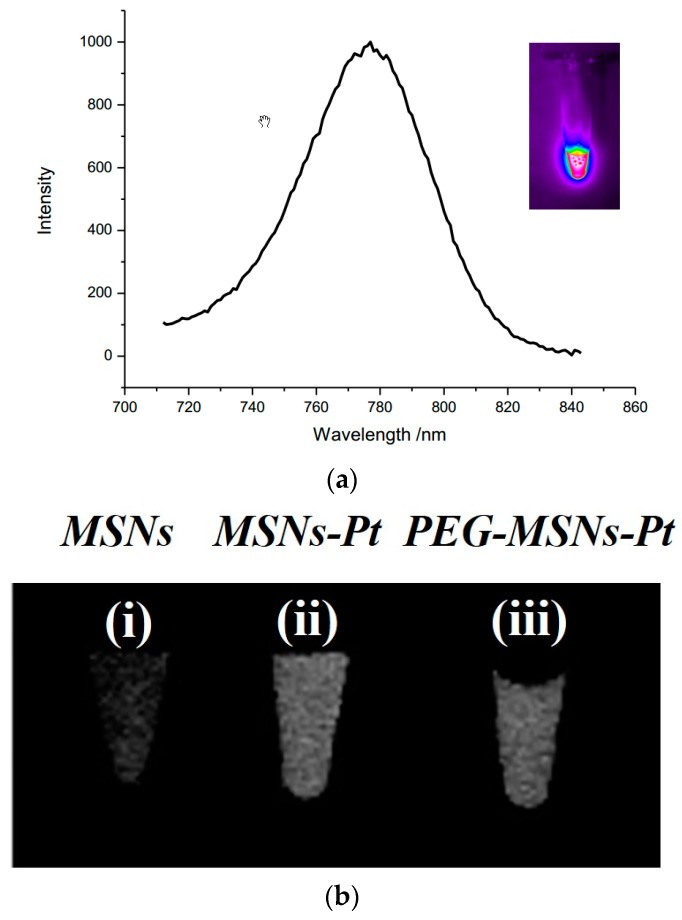
(**a**) The fluorescence spectrum of MSNs-Pt-Dy800 with excitation at 670 nm. The optical image in the inset shows MSNs-Pt-Dy800 with excitation at 770 nm and emission at 800 nm. (**b**) The computed tomography (CT) imaging of the tube array composed of various MSN samples: (i) MSNs, (ii) MSNs-Pt, and (iii) PEG-MSNs-Pt.

The optical properties of the MSNs-Pt-Dy800 are shown in Figure 4a. The fluorescence spectrum of MSNs-Pt-Dy800 was similar to that of Dy800, with the maximum emission at 783 nm. The optical image of MSNs-Pt-Dy800 sample was collected at an excitation of 770 nm, and pronounced NIR fluorescence was observed, as shown in the inset of Figure 4a. For the second modality, the CT imaging of the tube array composed of various MSNs samples is presented in Figure 4b. Compared to that of MSNs (Figure 4b-i), the CT contrast of MSNs-Pt (Figure 4b-ii) was much more pronounced. The PEG-MSNs-Pt still displayed an appreciable enhancement in CT contrast, as shown in Figure 4b-iii. These measurements reflect the active function of the dual modalities of MSNs-Pt-Dy800. With further development and validation, MSNs-Pt-Dy800 may serve as a potential tracer for cancer diagnostics in vivo.

**Figure 5:**
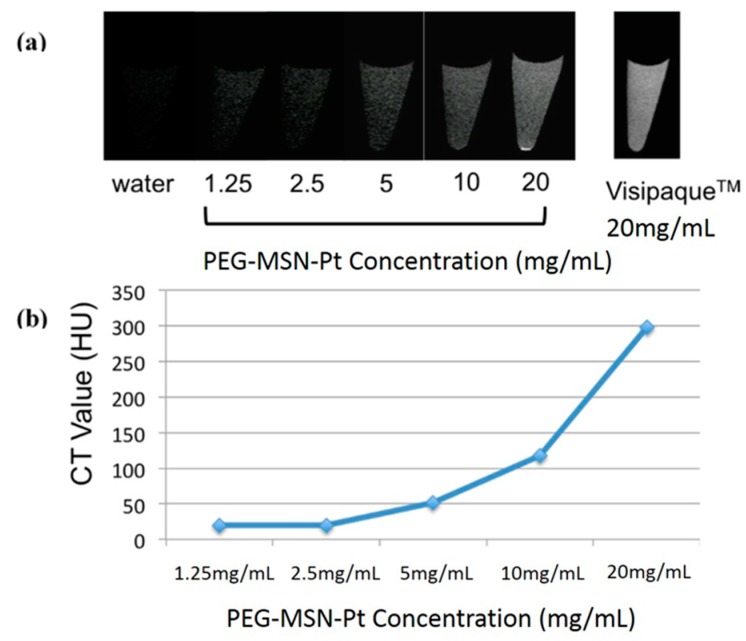
(**a**) In vitro CT images of PEG-MSNs-Pt at dosages of 1.25, 2.5, 5, 10, and 20 mg mL^−1^ and of the commercial CT contrast agent Visipaque^TM^ at a concentration of 20 mg mL^−1^. (**b**) The measured CT values of the PEG-MSNs-Pt nanoparticles (HU of Visipaque^TM^: 360).

The in vitro CT imaging of the tube array is illustrated in Figure 5a. As expected, the scans demonstrated a positive contrast enhancement in a dose-dependent manner. Appreciable enhancement for CT contrast was observed using 1.25 mg mL^−1^ of PEG-MSNs-Pt. The calculated CT signal contrast intensities within the range of 1.25~20 mg mL^−1^ of PEG-MSNs-Pt are plotted in Figure 5b. The CT signal using 20 mg mL^−1^ of PEG-MSNs-Pt (corresponding to 2.8 mg of Pt NPs) exhibited an intensity equivalent to that of the commercial iodine-containing CT contrast agent Visipaque^TM^ at 20 mg mL^−1^ (360 HU).

### 2.3. Biocompatibility and Cellular Uptake of MSNs-Pt NPs

**Figure 6:**
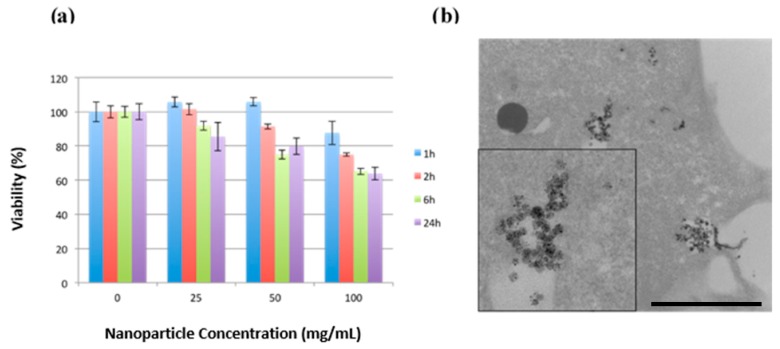
(**a**) Cell viability of PEG-MSNs-Pt NPs in MDA-MB-231 breast cancer cells. (**b**) A TEM image of MDA-MB-231 illustrates the cellular uptake of PEG-MSNs-Pt NPs after 24 h (Scale bar: 1 µm).

The in vitro cytotoxicity of the PEG-MSNs-Pt NPs was evaluated in MDA-MB-231 breast cancer cells using an MTT assay. The water-soluble PEG-MSNs-Pt NPs were tested at concentrations of 25, 50, and 100 µg mL^−1^ for incubation times of 1, 2, 6, and 24 h. The MTT assay (Figure 6a) showed that only minor cytotoxic responses were detected at concentrations below 50 µg mL^−1^ after 24 h of exposure. Furthermore, at the highest concentration of 100 µg mL^−1^, the cell viability decreased to 65% at 6 and 24 h. With regard to the cellular uptake, the TEM image of the cell section showed that the MSNs-Pt NPs were located in the endosomes after 24 h of incubation (Figure 6b). Some of the MSNs-Pt began to escape from the endosome to the cytoplasm (inset Figure 6b). Our results revealed high cell viability and cellular uptake of the MSNs-Pt NPs, which suggest that they possess excellent in vitro biocompatibility.

### 2.4. In Vivo Optical and CT Images of Dual-Modality MSNs

**Figure 7:**
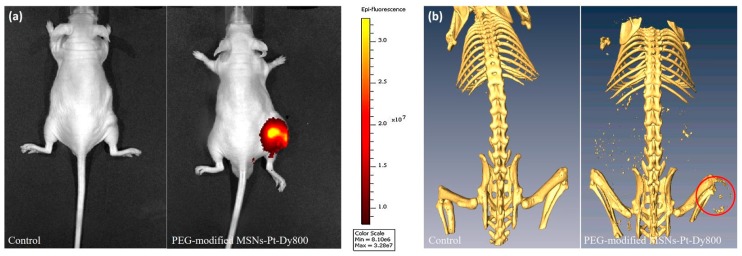
(**a**) An optical image of a mouse bearing the MDA-MB-231 tumor was obtained before (Control) and 24 h post-intratumoral injection of PEG-MSNs-Pt-Dy800. (**b**) CT images of a mouse bearing the MDA-MB-231 tumor (as indicated by the red circle) were taken before (Control) and 24 h after the intratumoral injection of PEG-MSNs-Pt-Dy800.

In vivo molecular imaging was performed in the MDA-MB-231 breast tumor animal model. PEG-MSNs-Pt (1.8 mg mL^−1^ Pt concentration, 150 μL) was injected into mice intratumorally. An OI image was acquired after injection (24 h), as shown in Figure 7a. Significant optical contrast was observed, and the NPs aggregated in the tumor site 24 h post-injection. On the other hand, significant CT contrast enhancement in the tumor was observed at 24 h, which might be due to the enhanced permeability and retention effect (red circle in Figure 7b). The CT contrast generated by the PEG-MSNs-Pt was similar to that of bone. The CT contrast of the PEG-MSNs-Pt was retained at 24 h post-injection. The PEG-MSNs-Pt exhibited great potential as a dual-modality molecular imaging contrast agent and can allow drug loading for therapeutics in future applications.

## 3. Discussion

PEG-MSNs-Pt-Dy800 showed a great contrast efficacy in OI/CT. Similarly, in previous reports, different sized FePt NPs were discussed, and their CT/Magnetic Resonance Imaging (MRI) contrast efficacies were compared. Smaller FePt NPs presented a higher concentration than large ones in mouse brains. The large FePt NPs exhibited longer circulation time in the blood, as well as a better contrast effect in CT/MRI [35]. These kinds of NPs possess great potential for translation of clinical diagnosis. Herein, we developed a dual-modality nanoplatform with enhanced contrasts for OI and CT to achieve the precision diagnosis of tumors in vivo. Such a nanoplatform consisted of nPtNPs reduced on the surface of MSNs and Dy800 loaded in the nanochannels of MSNs; further, PEG modification improved the circulation time in blood providing favorable pharmacokinetics. It allows us to efficiently perform fast tracing of NPs using optical imaging systems, followed by confirmation of orientation using CT.

It has been indicated that different hybrid NPs containing PtNPs possess contradictory effects, such as cell protection and cytotoxicity. It is worth noting that PtNPs exhibit high catalytic activity, scavenging the singlet oxygen and superoxide to protect cells [36,37,38]. On the other hand, nanoparticulate Pt (nano-Pt) showed cisplatin-like function to inhibit cancer cell progression, cytotoxicity and less drug resistance [41]. Previous reports also used microwave-assisted methods to synthesize mesoporous silica aerogel-supported PtNPs (SAP) for a proton exchange membrane fuel cell [42]. Similar to our design, a group reported nPtNPs within the nanochannel of mesoporous silica nanospheres as a catalyst for hydrolysis [43]. In this regard, we used MSNs with nPtNPs on the surface, which were much smaller than those reported previously. We suggest that nPtNPs on MSNs display a mechanism similar to anti-cancer platinum drugs, such as cisplatin. One important mechanism of cisplatin toxicity is increased oxidative stress. Cisplatin generates reactive oxygen species that induce cell death [44]. Among the series cell death pathway, the intracellular glutathione plays a crucial role in these mechanisms, as cisplatin-glutathione conjugates reduce the platinum binding to DNA and transport out of the cells protects dividing cells from cisplatin toxicity [45]. Besides the pronounced contrasts of dual-modality imaging, in our study, we also attempted to monitor the induced cytotoxic effects involving intracellular glutathione (GSH) and related enzymes’ variations after treatment with MSNs-Pt. Our preliminary results implicated that MSNs-Pt have much higher therapeutic efficacy than PEG-MSNs-Pt in both in vitro and in vivo experiments. Likewise, it was demonstrated that nano-Pt triggered the autophagy and immunological responses in the tumor area [41]. These preclinical experiments are currently ongoing; we are focusing on the underlying molecular mechanisms of therapeutic effects rendered by MSNs-Pt with the nPtNP distributions on the different topological domains of MSNs, i.e., nPtNPs reduced on the MSN surface and surface/channels, etc.

In summary, we reported on the microwave synthesis of nPtNPs on the surface of NIR dye-doped MSNs for dual-modality imaging of OI and CT. The PEG-stabilized NPs were very stable in aqueous solution and did not affect cell viability; furthermore, they provided an active surface zone for further conjugation of the targeting motif. The CT contrast was significantly enhanced following the integration of naked nPtNPs on MSNs. The composite NPs have great potential for use as probes in optical and CT imaging and for use as drug delivery carriers due to the features of MSNs. These dual-modality NPs provide significant CT and optical contrast, and they remained in the tumor for at least 24 h. The novel microwave synthesis of MSNs-Pt provides a simple and rapid approach for the production of nanoparticle-based OI/CT dual-modality contrast agents.

## 4. Materials and Methods

### 4.1. Chemicals

Tetraethyl orthosilicate (TEOS), ammonium hydroxide (30%), and ethanol were purchased from Acros (Acros Organics, Geel, Belgium). (3-Aminopropyl)trimethoxysilane (APTMS), *n*-octane, and chloroplatinic acid hexahydrate (H_2_PtCl_6_) were obtained from Sigma Chemical Co. Dylight800 NHS ester was purchased from Pierce. Methyl-PEG-Thiol MW5000 was obtained from NanoCS (Nanocs Inc., New York, NY, USA).

### 4.2. Preparation of MSNs-NH_2_ NPs

The large-pore MSNs-NH_2_ sample with a sphere-like, well-ordered pore structure was synthesized using a low concentration of TEOS, a surfactant, and a basic (NH_4_OH) catalyst in a two-step preparation. The sol-gel process for the co-condensation of TEOS and the synthesis of the MSNs-NH_2_ sample is described as follows. First, cetyltrimethylammonium bromide (CTAB) (0.58 g) was dissolved in NH_4_OH (0.51 M, 300 mL) at 50 °C, and then 4.5 g of n-octane was added under vigorous stirring. Then, dilute TEOS (0.2 M in 5 mL of ethanol) was added to the solution. After the solution was stirred for 5 h, APTMS (12% *v*/*v* in 5.0 mL of ethanol) and TEOS (1.0 M in 5.0 mL of ethanol) were added under vigorous stirring for an additional 1 h. The solution was aged at 40 °C for 24 h. Samples were collected by centrifuging at 12000 rpm for 20 min, washed, and re-dispersed with deionized water and ethanol several times. The solid products were obtained by centrifuging. The surfactant templates were removed by extraction in acidic ethanol (approximately 1.0 g of HCl in 50 mL of ethanol at 65 °C for 24 h).

### 4.3. Synthesis of Platinum NPs on the MSNs

The adsorption of Pt^4+^ ions on the surfaces of the aminosilane-modified silica nanoparticles was achieved by harnessing electrostatic attractions between the positively charged surface of the amine-modified silica and the negative charge of the PtCl^6−^ ions. The strong attractions between the ions in each ion pair (–NH^3+^ PtCl^6−^–) provided stability for the further reduction of the nPtNPs on the surfaces of the silica nanoparticles. The loading solution was mixed by adding 50 mg of MSNs-NH_2_ with 50 µL of H_2_PtCl_6_ solution (1 mg mL^−1^) in the presence of 50 mL of water. The mixture was stirred at room temperature for 0.5 h. The resulting MSNs-NH^3+^ PtCl^6−^ complexes were washed twice with pure water and then centrifuged at 11000 rpm for 20 min. The MSNs-NH^3+^ PtCl^6-^ complexes were reduced by re-suspending the above MSNs-NH^3+^ PtCl^6−^ complexes in 5 mL of H_2_O; subsequently, the solution was exposed to a 300 W fixed-mode microwave synthesis system (Discover SP-D, CEM Tech., Matthews, NC, USA) for 10 min at 120 °C. The MSNs-Pt were collected by centrifuging at 12000 rpm for 20 min, washed, and re-dispersed with deionized water and ethanol several times. The platinum concentration was measured by inductively coupled plasma mass spectrometry.

### 4.4. Preparation of MSNs-Pt-Dy800 Samples

The covalent conjugation of the near-infrared fluorophore DyLight in the MSNs-Pt nanochannels to obtain MSNs-Pt-Dy800 was conducted as follows. First, a Dy800-NHS ester stock solution was prepared by mixing Dy800-NHS ester powder (1 mg) in Dimethylformamide (DMF) (1 mL). Next, 200 μL of the DMF/Dy800 stock solution was added to an extracted MSNs-Pt sample (15 mg), and the mixture was further diluted in 50 mL of DMF. After stirring at room temperature for 2 h, the MSNs-Pt-Dy800 complex was washed twice with Ringer’s solution, followed by centrifuging for 20 min at 12000 rpm. The amount of Dy800 loaded in the individual MSNs-Pt samples was determined by measuring the decrease in optical absorption of the residual solution at 777 nm.

### 4.5. PEG Conjugation

First, 100 mg of MSNs-Pt-Dy800 nanoparticles was added to a flask in the presence of 20 mL of a mPEG5000-SH solution (1 mg mL^−1^). After stirring the solution for 20 min at room temperature, the solution was centrifuged, washed three times with water, and suspended in 10 mL of water for in vitro experiments.

### 4.6. Characterization

The ζ-potentials of all the samples were measured using a Malvern Zetasizer 3000 NANOZS (Westborough, MA, USA). The zeta potential distribution was obtained from the average of ten measurements. The morphologies of the samples were characterized by TEM (Hitachi H-7650 operating at an accelerating voltage of 80 kV, Berkshire, UK). Thermogravimetric analysis (TGA) data were obtained with a NETZCH TG209-F3 thermogravimetric analyzer (NETZCH, Germany). The samples were heated from 30 to 650 °C at a heating rate of 15 °C per minute under nitrogen. The Pt content in the samples was measured using inductively coupled plasma mass spectrometry (ICP-MS, PerkinElmer, Akron, Ohio, USA). The sample preparation was as follows: 100 μL of MSN-Pt NP samples was placed in a TFM digestion vessel (CEM Corporation, Matthews, NC, USA). Then, a mixture of 0.5 ml of HNO_3_ and 1.5 ml of HCl was added. The sample was heated to 200 °C via microwave irradiation for 20 min. The samples were then transferred to polypropylene (PP) vials and diluted 200 times.

### 4.7. Cell Viability Assay

The cell viability in the presence of the NPs was evaluated using a 3-[4-dimethylthiazol-2-yl]-2,5-diphenyltetrazolium bromide (MTT, Sigma, MO, USA) assay. For the MTT assay, MDA-MB-231 cells were seeded in 96-well plates at a density of 1 × 10^4^ per well in 200 μL of media and grown overnight. The cells were then incubated with various concentrations of PEG-MSNs-Pt (0, 25, 50, and 100 μg mL^−1^) for 1, 2, 6, and 24 h. Following this incubation, the cells were incubated in media containing 0.1 mg mL^−1^ of MTT for 1 h. Then, the MTT solution was removed, and the precipitated violet crystals were dissolved in 200 μL of dimethyl sulfoxide (DMSO). The absorbance was measured at 560 nm using a microplate reader.

### 4.8. Cellular Uptake and TEM Imaging

To determine the cellular uptake of nanoparticles, MDA-MB-231 cells were plated in 12-well plates pre-coated with poly-d-lysine containing complete media and 10% FBS at a density 1 × 10^4^ cells/well. The dishes were then placed in an incubator overnight at 37 °C and 5% CO_2_ to permit cell attachment. The next day, the cells were carefully washed with phosphate-buffered saline (PBS) and refreshed with 3 mL of serum-free medium. Nanoparticles were added to the well and mixed gently at a final concentration of 50 μg mL^−1^. The treated cells were then incubated at 37 °C and 5% CO_2_ for 6 h. After incubation, the cells were rinsed three times with PBS.

For cell ultra-sections, the treated cells were pre-fixed in 2.5% glutaraldehyde for 1 h and then washed twice with 0.1 M PBS (pH 7.0). Osmium tetroxide (2%) was used for 1 h for post-fixation. Dehydration was then performed using an ascending series of ethanol concentrations at 30%, 50%, 70%, 80%, and 95% for 10 min each, followed by three 100% ethanol dehydrations for 10 min each. The dehydrated samples were then embedded in Spurr’s resin and allowed to polymerize for 15 h at 68 °C. Ultrathin sections were cut with a Leica UC6 ultramicrotome and examined with a Hitachi H-7650 TEM at an accelerating voltage of 80 kV without further staining.

### 4.9. In Vivo Optical Imaging

In vivo fluorescence imaging was carried out on a commercial Xenogen/Caliper IVIS-200 In Vivo Imaging System (with isoflurane anesthesia, PerkinElmer, Akron, OH, USA) and on a home-made in vivo optical imaging system (with urethane anesthesia). For the latter, light from a 100 W Hamamatsu xenon lamp was passed through fluorophore-dependent bandpass interference filters (Model 545AF75, Omega Optical Inc., Brattleboro, VT, USA) to induce Dy800 fluorescence. The imaging sensor was a charge-coupled device (CCD) camera (DW436, Andor Technology Ltd, Belfast, Northern Ireland) that was thermoelectrically cooled to −90 °C. Bandpass interference filters (FF495 Ex 02-25, Smerok, New York, NY, USA) were placed over a 50 mm f/1.2 lens (Nikon, Tokyo, Japan) to allow fluorescence to pass but to block the excitation light. The animal body temperatures were maintained at 37 °C via a heating pad. Animals used in these studies were approved under the guidelines of the Institutional Animal Care and Use Committee (IACUC) of the National Health Research Institutes (Animal protocol number: NHRI-IACUC-104012A, 6 February 2015).

### 4.10. CT Imaging

For the in vitro studies, 10 mg mL^−1^ of the PEG-MSNs-Pt NPs were prepared. The prepared solutions were placed in 0.25 mL tubes to make concentrations of 1.25, 5, 10, and 20 mg mL^−1^. Images were then obtained using an animal CT scanner (Trumph X, Gamma Medica Ideas, Northridge, CA, USA) with the following parameters: tube voltage, 70 kV; current intensity, 180 μA; and field of view, 512 × 512 mm^2^. For the animal studies, 1 × 10^6^ MDA-MB-231 cells were subcutaneously injected into female nude mice. During the imaging, the mice were anesthetized with 2% isoflurane, and a pre-contrast CT scan was performed using the same setting as in the phantom study. Then, the MSNs-Pt NPs (10 mg mL^−1^, 100 μL) were injected intratumorally, and images were sequentially obtained from 30 min to 24 h post-injection.
